# Special Treatments of Consumption

**Published:** 1913-08-09

**Authors:** 


### SPECIAL TREATMENTS OF CONSUMPTION.

### The Continental Expedient of Rib-cutting.

The generalisation may be ventured, in spite of Matthew Arnold's warning to the critic against all forcible "non-central" expressions of opinion, that past and present treatments of consumption—we mean special treatments other than the sanatorium—fall into three classes: a huge one and two tiny ones. The first comprises all the inhalations, drugs, and countless other medicaments put forward just as confidently, just as naïvely, now as they were two thousand years ago. Then it was snails or sulphur; now it is something with a fancy name; but the effect is just the same, being at most moral and therefore transient. In the second must be put intensive tuberculinisation; the therapeutic value of this is nothing to boast of, but in all probability it *is* positive—considering laboratory experiment and the comparative clinical statistics gathered abroad—and, of course, there is also the promise of considerable advance. The third special treatment is lung-collapse. Only such cases are suitable for this measure as have one sound or soundish lung to get along with while the other is being compressed and the disease in it stifled. Moreover, as a new treatment lung-collapse is only justifiable when ordinary sanatorium measures look like failing. At present we find it applicable in only 5 or 6 per cent., although, as experience mounts up, this low proportion is likely to rise. What is clearly indicated, then, as the phthisiotherapist's aim, is to secure to as many consumptives as possible the unprecedented benefits lung-collapse can bring.

Now one obstacle has been the following: If you want to compress a patient's lung the first, because the least serious, measure to be tried is the induction of artificial pneumothorax, putting nitrogen gas, that is, between the two layers of the pleura, between the chest wall and the lung, between, as it were, the outer cover and the inner tube, and so flattening the latter. But what unfortunately sometimes happens is (and the condition cannot be foretold) that the two layers aforesaid are stuck together; chronic dry pleurisy has caused adherent pleura; patches on the tube have stuck to the cover as well. It follows that no gas can get in and the lung cannot be compressed and collapsed.

### A Continental Expedient.

The expedient some Continental surgeons* then resort to, an expedient which will be dealt with at some length by Professor Sauerbruch this week at the International Congress, forms the subject of the present remarks. They cut out small pieces of several ribs, generally behind only, near the spine, sometimes in front only, near the breast bone, sometimes (a severe procedure) both in front and behind, and the collar bone as well, thus doing away with inspiratory movement and allowing the elastic force of the lung to collapse that organ.

It is regrettable that no work on this subject in its modern developments seems to have been done at our large consumption hospitals, the reason being that as regards the initial measure, artificial pneumothorax, other countries are much ahead of our own. But British tuberculosis workers are certain to follow in the footsteps of their foreign confrères. The therapeutic effect of lung compression in well selected cases is too striking, and even when only lasting a few months, the great diminution in sputum forms, as has been pointed out, a highly desirable sanitary measure. Lastly, but certainly not least, no expensive apparatus is necessary. With the wide public interest now being shown in pulmonary tubercle every tuberculosis officer in charge of beds for advanced cases should have some knowledge of Forlanini's discovery. And those who put in serious work at artificial pneumothorax, it is safe to predict, will find themselves chafing when confronted with adherent or partially adherent pleura. In one case gas has been successfully got in; the temperature falls, the sputum dwindles to nothing, the patient gets up. The whole outlook is changed. In another no way can be found, the manometer remaining motionless; the *descensus averni* goes on all the quicker because of hope disappointed. Or around an old cavity the apex of the lung is closely bound to the ribs, and only the base can be compressed; when, although partial benefit accrues, what bars real success is the non-collapse of the cavitous caseous area. Such contrasts should make those workers who encounter them study very carefully the meritorious article mentioned in the footnote.

### Indication for Rib-cutting.

What is the indication for the cutting operation? This single one; failure, through adherent pleura, to induce complete artificial pneumothorax. What the results—and in cases, be it remembered, going downhill? The results of forty-one cases are: eight cured and seven more expected to be cured among the much improved and improved classes, which number seven and thirteen respectively; unchanged three; made worse four; dead six (only one on the table). No great surgical armamentarium seems needed. At this Zürich clinic the apparatus for obtaining artificial atmospheric pressures was held in readiness but never required. In nearly all instances local anæsthesia sufficed, even when resection of the first rib was undertaken. It is stated that not much bony deformity results. But, different from artificial pneumothorax, it must be noted that a lung thus collapsed is collapsed for good; there can necessarily be no leaving off of compression after a year or so and allowing re-expansion. The sphere of this operation, therefore, seems strictly limited to rather desperate cases, a fact which is worth mentioning,

* "Die Extrapleurale Thorakoplastik": von Sauerbruch und Elving in *Ergebnisse der inneren Medizin u. Kinderheilkunde*, Bd. X., 1913.

because the sphere of artificial pneumothorax will probably soon be extended to earlier, less severe ones, provided only the disease shows some unilaterality. Granted, unilateral cases may be seen which have been cured by hygienic-dietetic means alone. Nevertheless, relapse being as it is the chief defect of sanatorium treatment, it would seem as well, now that the induction of artificial pneumothorax is so safe in well instructed hands, to apply every possible therapeutic measure at the earliest possible date. But at present, at any rate, procedures like extra-pleural thoracoplasty or division of one phrenic nerve seem emergency measures only. It seems so at present; but we venture to predict that the treatment of pulmonary tuberculosis will be marked by the effective and permanent incursion of the surgeon, nearly as much as the treatment of many other diseases have been.

